# The Effects of Almonds on Gut Microbiota, Glycometabolism, and Inflammatory Markers in Patients with Type 2 Diabetes: A Systematic Review and Meta-Analysis of Randomised Controlled Trials

**DOI:** 10.3390/nu13103377

**Published:** 2021-09-26

**Authors:** Omorogieva Ojo, Xiao-Hua Wang, Osarhumwese Osaretin Ojo, Amanda Rodrigues Amorim Adegboye

**Affiliations:** 1 Faculty of Education, Health and Human Sciences, School of Health Sciences, University of Greenwich, Avery Hill Campus, Avery Hill Road, London SE9 2UG, UK; 2 The School of Nursing, Soochow University, Suzhou 215006, China; wangxiaohua@suda.edu.cn; 3 South London and Maudsley NHS Foundation Trust, University Hospital, Lewisham High Street, London SE13 6LH, UK; osarhumwese.ojo@slam.nhs.uk; 4 Faculty of Health and Life Sciences, School of Nursing, Midwifery and Health, Coventry University, Priory Street, Coventry CV1 5FB, UK; ad6287@coventry.ac.uk

**Keywords:** type 2 diabetes, almonds, tree nuts, glycated haemoglobin, gut microbiota, body mass index

## Abstract

The use of nutritional interventions for managing diabetes is one of the effective strategies aimed at reducing the global prevalence of the condition, which is on the rise. Almonds are the most consumed tree nut and they are known to be rich sources of protein, monounsaturated fatty acids, essential minerals, and dietary fibre. Therefore, the aim of this review was to evaluate the effects of almonds on gut microbiota, glycometabolism, and inflammatory parameters in patients with type 2 diabetes. Methods: This systematic review and meta-analysis was carried out according to the preferred reporting items for systematic review and meta-analysis (PRISMA). EBSCOhost, which encompasses the Health Sciences Research Databases; Google Scholar; EMBASE; and the reference lists of articles were searched based on population, intervention, control, outcome, and study (PICOS) framework. Searches were carried out from database inception until 1 August 2021 based on medical subject headings (MesH) and synonyms. The meta-analysis was carried out with the Review Manager (RevMan) 5.3 software. Results: Nine randomised studies were included in the systematic review and eight were used for the meta-analysis. The results would suggest that almond-based diets have significant effects in promoting the growth of short-chain fatty acid (SCFA)-producing gut microbiota. Furthermore, the meta-analysis showed that almond-based diets were effective in significantly lowering (*p* < 0.05) glycated haemoglobin (HbA1c) levels and body mass index (BMI) in patients with type 2 diabetes. However, it was also found that the effects of almonds were not significant (*p* > 0.05) in relation to fasting blood glucose, 2 h postprandial blood glucose, inflammatory markers (C-reactive protein and Tumour necrosis factor α, TNF-α), glucagon-like peptide-1 (GLP-1), homeostatic model assessment of insulin resistance (HOMA–IR), and fasting insulin. The biological mechanisms responsible for the outcomes observed in this review in relation to reduction in HbA1c and BMI may be based on the nutrient composition of almonds and the biological effects, including the high fibre content and the low glycaemic index profile. Conclusion: The findings of this systematic review and meta-analysis have shown that almond-based diets may be effective in promoting short-chain fatty acid-producing bacteria and lowering glycated haemoglobin and body mass index in patients with type 2 diabetes compared with control. However, the effects of almonds were not significant (*p* > 0.05) with respect to fasting blood glucose, 2 h postprandial blood glucose, inflammatory markers (C-reactive protein and TNF-α), GLP-1, HOMA–IR, and fasting insulin.

## 1. Introduction

The use of nutritional interventions is one of the strategies for managing diabetes, which is on the increase worldwide. It is projected that the global prevalence of diabetes could reach 700 million by 2045, up by 51% from 463 million who were living with the condition in 2019 [1]. Over 90% of people with diabetes have type 2 diabetes, which is linked to lifestyle factors [2], and this has implications in terms of morbidity and mortality. Poor diabetes control increases the costs of healthcare as a result of potentially avoidable hospital treatment and drug prescription and in the UK, the total annual spending on patients with type 2 diabetes is expected to rise to about £2.2 billion by 2040–2050 [3,4]. Therefore, nutritional interventions, which are effective in terms of clinical outcomes, are often recommended for diabetes management [5]. In this regard, the use of nuts, including tree nuts, such as almond, walnut, hazelnut, cashew nuts and Brazil nuts, and groundnuts (mainly peanuts), which are high in unsaturated fatty acids and are rich sources of bioactive nutrients that have significant metabolic and cardiovascular health benefits, have been suggested [6,7].

Almonds are the most consumed tree nut and they are known to be rich sources of protein, monounsaturated fatty acids, essential minerals, and dietary fibre [6,8]. The role of dietary fibre in modulating gut microbiota dysbiosis and in the regulation of glycaemic parameters have been demonstrated in previous systematic reviews and meta-analyses [9,10] and in randomised controlled trials [11,12].

### 1.1. Description of the Intervention

Nuts have been part of the human diet for centuries. Nuts are included in different recipes and, more recently, nuts, particularly almonds, have been consumed as a healthy snack [13]. However, the level of consumption of nuts may vary globally, across different populations. Almonds are tree nuts that have a low glycaemic index, are rich in dietary fibre and unsaturated fatty acids, and have low carbohydrate content [6]. The macro- and micronutrient components of almonds, including monounsaturated fatty acids, polyunsaturated fatty acids, fibre, vitamins, minerals, phytosterols, and polyphenols, have been associated with health benefits including anti-inflammatory and lipid-lowering properties [6,8]. Almonds also have antioxidant properties [8]. The polyphenols and fibre content of almonds may be used as substrates for gut microbial growth and regulation of gut microbiota [8]. It has been suggested that there is an inverse relationship between the consumption of nuts and the risk of developing type 2 diabetes [6].

### 1.2. How This Intervention Might Work

It has been reported that almond consumption increases satiety, decreases postprandial glycaemia, and regulates oxidative stress [6]. Almond consumption may also decrease the rate of nutrient digestion, reduce glucose response, and stimulate incretin and the production of glucagon-like peptide- 1 (GLP-1) [6,14]. The fermentation of the dietary fibre component of almonds may lead to improvement in the composition and metabolic products of gut microbiota, such as an increase in the prevalence of health-promoting bacteria and short-chain fatty acid production, including propionic, butyric, and acetic acid [10,15,16]. The short-chain fatty acids produced during this process have been shown to improve glycometabolism in patients with diabetes [10,15,17]. An almond-based low-calorie diet has also been found to be effective in reducing weight [18], which is useful in promoting insulin sensitivity and regulating glycaemic control.

### 1.3. Why It Is Important to Do This Review

Incorporating almonds in well-balanced healthy diets have been shown to confer beneficial effects on glycaemic control in patients with type 2 diabetes [6,14,19]. However, it would appear that previous systematic reviews and meta-analyses in this area of research have either focused on the effects of tree nuts in general [20,21,22], on blood pressure [23], or on fasting blood lipids [24]. For example, Mohammadifard et al. [23], conducted a systematic review and meta-analysis on the effect of tree nuts, peanuts, and soy nuts on blood pressure, while Blanco-Mejia et al.’s [20] review focused on the effects of tree nuts on metabolic syndrome. Muley et al. [21], on the other hand, evaluated the effects of tree nuts on glycaemic control in adults with type 2 diabetes, while Musa–Veloso et al. [24] examined the effects of almond consumption on fasting blood lipids. Viguinliouk et al.’s [22] review examined the effect of tree nuts on glycaemic control in patients with diabetes.

However, the present systematic review and meta-analysis will complement the existing literature by providing evidence that focuses on the role of almonds on gut microbiota, glycaemic control, and inflammatory markers. There are indications that increased markers of inflammation and disequilibrium of the gut microbial community are associated with the dysregulation of glycaemic control and type 2 diabetes [10,25,26].

### 1.4. Aim

To evaluate the effects of almonds on gut microbiota, glycometabolism, and inflammatory parameters in patients with type 2 diabetes.

## 2. Methods

This systematic review and meta-analysis was carried out according to the preferred reporting items for systematic review and meta-analysis (PRISMA) [27].

### 2.1. Types of Studies

Only randomised controlled trials (RCTs) including crossover and parallel designs were included in this review.

### 2.2. Types of Participants

Adult participants with type 2 diabetes regardless of the existence of co-morbidities (e.g., obesity) were selected for the review.

### 2.3. Types of Interventions

We included RCTs comparing the provision of almonds or advice to increase almond consumption with a control group (no intervention or habitual diet or other types of nuts) also with type 2 diabetes. There was no restriction regarding the minimum and maximum amount of almonds consumed. RCT including multiple interventions (diet and exercise) were not considered. There was no restriction regarding the duration of the interventions.

### 2.4. The Inclusion Criteria

Randomised controlled trials involving participants with type 2 diabetes and aged 18 years and over were included in this review. Studies with outcomes of interest involving gut microbiota, glycometabolism, anthropometric measurements, and inflammatory parameters were also included in this review.

### 2.5. The Exclusion Criteria

Studies excluded were those with prediabetes; involving other tree nuts other than almonds, such as walnuts; involving patients with gestational diabetes, type 1 diabetes, and only healthy populations. Cluster randomised trials were not eligible for inclusion. Furthermore, studies with participants aged below 18 years and animal studies were excluded from this review. Pregnant and lactating women were not included.

### 2.6. Types of Outcome Measures

The following were the primary outcome measures of interest:

- Gut microbiota;
- Blood glucose parameters: glycated haemoglobin (HbA1c, %);
- Inflammatory markers: tumour necrosis factor α (TNF-α); high-sensitivity C-reactive protein (hsCRP);
- Body mass index (BMI) (Kg/m^2^).
- Secondary outcome measures of interest:
- Fasting blood glucose (FBG, mmol/L);
- Postprandial blood glucose (2 h PBG, mmol/L);
- Homeostatic model assessment of insulin resistance (HOMA–IR);
- Glucagon-like peptide-1 (GLP-1);
- Fasting insulin.

### 2.7. Search Methods for Identification of Studies

EBSCO-host (which encompasses the Health Sciences Research Databases, including MEDLINE, Academic Search Premier, APA PsycInfo, Psychology and Behavioural Sciences Collection, APA PsycArticles databases, and CINAHL Plus with Full Text), Google Scholar, and EMBASE were the databases searched for relevant articles. In addition, the reference lists of articles were also searched based on the population, intervention, control, outcome, and study (PICOS) framework (Table 1). Searches were carried out from database inception until 1st August 2021. Search terms were drawn from medical subject headings (MesH) and synonyms and were combined using Boolean operators (OR/AND). Two members of the research team (O.O. and O.O.O.) conducted the searches independently and these were cross-checked by the other two members of the team (X.W. and A.R.A.A). Resolution of differences was through discussion and consensus. Search results from databases were exported to EndNote (Analytics, Philadelphia, PA, USA) and de-duplicated.

## 3. Data Collection and Analysis

### 3.1. Selection of Studies

**Figure 1:**
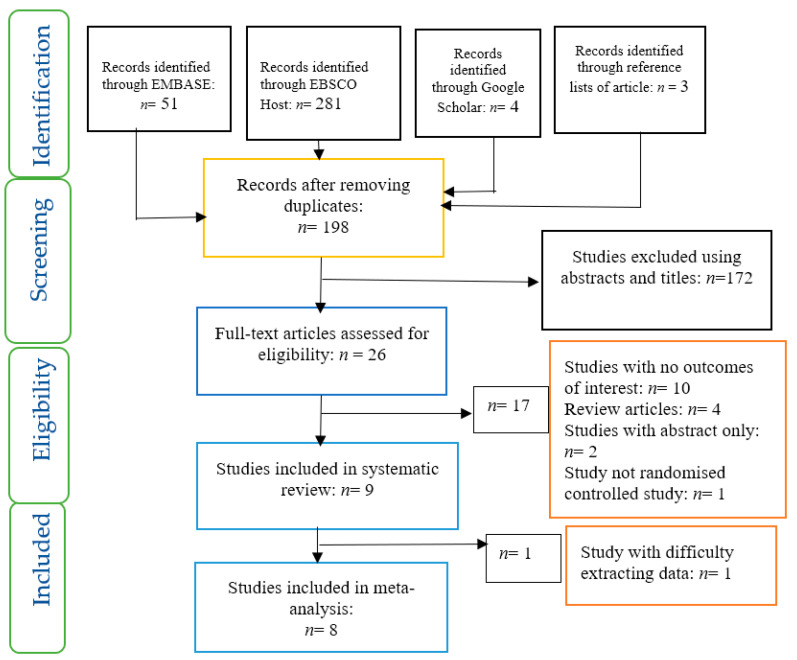
PRISMA flow chart on selection and inclusion of studies.

The PRISMA flow chart (Figure 1) was based on a set of inclusion and exclusion criteria that were used to select the studies included.

### 3.2. Data Extraction and Management

The data were extracted in a pre-piloted and standardised form. We extracted the following information: the country where the study was conducted, characteristics of the study population (e.g., mean age), sample size, outcome data, intervention details (duration) (Table 2).

Where the findings of more than one study were reported in one article, only the data from the study pertaining to patients with diabetes were included in the analysis.

The data was extracted by one researcher (O.O.) from the articles included and the three other members of the research team (O.O.O., X.W., A.R.A.A) cross-checked the information. Final values and changes from baseline were used to compare the intervention group with the control group. The units of measurements for some of the parameters were converted to ensure the same unit of measurements for all the studies for that parameter. In studies reporting values in median and 1st and 3rd quartile values, these were converted to means and standard deviations.

### 3.3. Assessment of Risk of Bias in Included Studies

Two members of the research team (O.O. and O.O.O.) evaluated the risk of bias of the included studies using the domain-based risk assessment tool [28]. The results were cross-checked by the other two members of the team (X.W. and A.R.A.A). Allocation concealment, the random sequence generation, blinding of outcome assessment, blinding of participants and personnel, selective reporting, incomplete outcome data, and other biases were the domains evaluated [29].

The risk assessment was conducted using the Review Manager 5.3 software (Copenhagen, Denmark) [28].

### 3.4. Data Analysis

Whenever there were enough trial reporting data on the same outcome, we performed a meta-analysis. Continuous data were analysed as mean difference (MD) with 95% confidence intervals (CIs), except for the fasting insulin due to differences in the units of measurements of the studies included and, thus, the standardised mean difference (SMD) was used for the meta-analysis. Forest plots were used to depict the results of the meta-analysis and in respect of statistical significance of the overall effect of the intervention, this was set at *p* < 0.05.

Sensitivity analysis was also conducted by removing studies one by one from the meta-analysis to assess the level of consistency of the results. The *I*^2^ statistic expressed as percentage was used to measure the degree of heterogeneity of studies included [29] in the review. A fixed-effects model was used for the meta-analysis for all the parameters of interest except for the fasting insulin due to differences in the units of measurements of the studies included and the standardised mean difference was used for the meta-analysis. Whenever a substantial heterogeneity (≥50%) was observed and there were enough studies included in the outcome, subgroup analysis was conducted. In addition, final values and changes from baseline were used to compare the intervention group with the control group [29]. If 10 or more studies were included, we would have performed a funnel plot to assess the presence of publication bias and small study effect. The meta-analysis was carried out in Review Manager (RevMan) 5.3 software [28].

## 4. Results

Nine studies were included in the systematic review and eight were used for the meta-analysis (Figure 1). The description and characteristics of eligible studies, including the type of study, details of sample, mean age, the aim of study, interventions, and results are outlined in Table 2. While one study was conducted in Canada [30], three each were conducted in Taiwan [31,33,34] and the USA [32,35,36], and two in China [6,14].

### 4.1. Evaluation of the Risk of Bias of Included Studies

**Figure 2:**
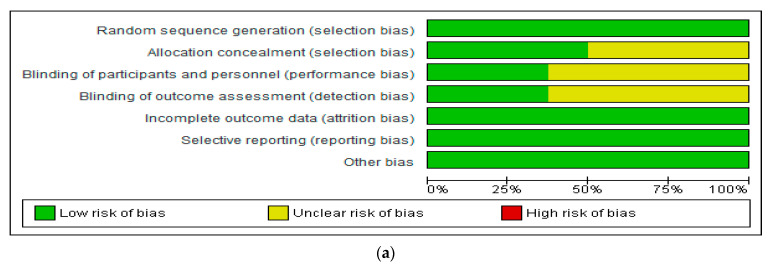

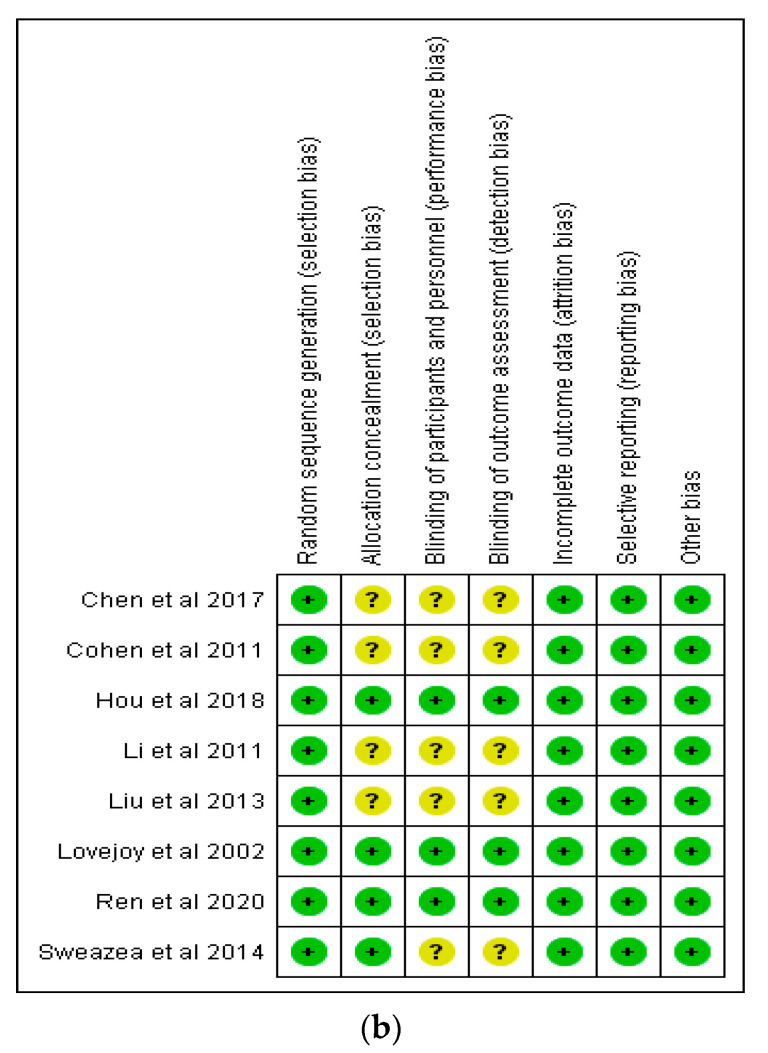
Shows (**a**) risk of bias graph and (**b**) risk of bias summary of studies included.

The risk of bias of included studies is shown in Figure 2a,b. All studies showed a low risk of bias in relation to the random sequence generation (selection bias), incomplete outcome data (attrition bias), and selective reporting (reporting bias). However, unclear risk of bias was found in relation to allocation concealment, blinding of participants and personnel, and blinding of outcome assessments in some of the studies [31,32,33,34,36].

The presentation of the results of the systematic review and meta-analysis were divided into.

Gut microbiota, glycaemic control, inflammatory parameters, body mass index, homeostatic model assessment of insulin resistance (HOMA-IR), glucagon-like peptide-1 (GLP-1), and fasting insulin.

### 4.2. Gut Microbiota

Only one study [14] examined the effects of almonds on gut microbiota. Ren et al. [14] found that the almond-based low-carbohydrate diet (LCD) significantly increased the short-chain fatty acid (SCFA)-producing bacteria *Roseburia, Ruminococcus,* and *Eubacterium*. In particular, the LCD group had a significantly higher population of *Roseburia* (*p* < 0.01) at the genus level compared with the low-fat diet (LFD) group by the third month, and compared to the baseline, *Eubacterium* (*p* < 0.01) and *Roseburia* increased significantly (*p* < 0.05) and *Bacteroides* (*p* < 0.05) significantly decreased in the almond-based LCD group.

### 4.3. Glycaemic Control

Chen et al. [31] did not find any significant effect with respect to change in glycated haemoglobin (HbA1c) and fasting serum glucose values in the almond-based and control diets. However, in the study by Cohen et al. [32], there was a significant reduction (*p* = 0.045) in HbA1c in the almond-based diet group compared with control group. Ren et al. [14] also showed that almond-based LCD may be effective in modulating glycometabolism in patients with diabetes.

Bodnaruc et al. [30] noted that the almond-based meal was associated with lower postprandial glucose. According to Hou et al. [6], while the almond-based diet did improve fasting, postprandial blood glucose, and glycated haemoglobin in patients with type 2 diabetes, these were not significantly different from the control group. Li et al. [33] observed that including almonds in a healthy diet led to significant improvement (*p* < 0.05) in glycaemic control, while Lovejoy et al. [35] showed that an almond-enriched diet had no significant effect (*p* > 0.05) on glycaemia in patients with diabetes.

**Figure 3:**
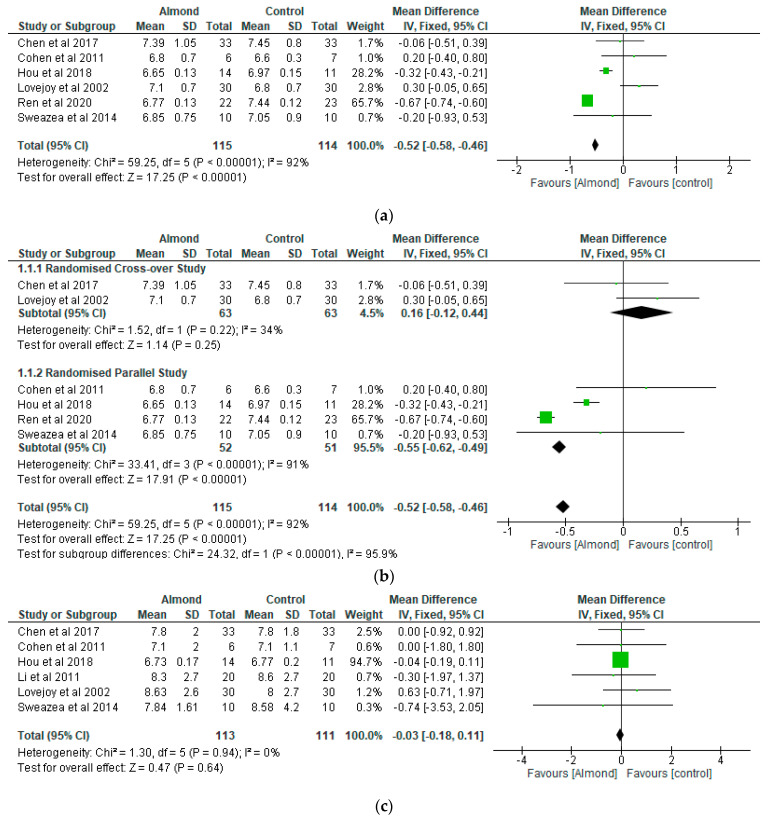

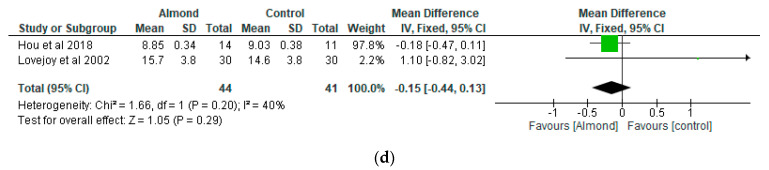
The effect of almonds on (**a**) glycated haemoglobin (Hba1c, %), (**b**) Hba1c (%)—subgroup analysis; (**c**) fasting blood glucose (mmol/L); (**d**) 2 h postprandial blood glucose (mmol/L).

With respect to the meta-analysis, the results of the effects of almonds on glucose control are shown in Figure 3a–d. Six studies each contributed data for the HbA1c analysis (almond group (gp), *n* = 115; control gp, *n* = 114) (Figure 3a; sub-group analysis, Figure 3b) and fasting blood glucose analysis (almond gp, *n* = 113; control gp, *n* = 111) (Figure 3c). The almond-based diet group experienced a significant reduction (*p* < 0.001) in HbA1c levels compared to the control group with a mean difference of −0.52 (95% CI: −0.58, −0.46). Regarding the 2-hour postprandial blood glucose levels, two studies contributed to the data analysis (almond gp, *n* = 44; control gp, *n* = 41) (Figure 3d). The levels of fasting blood glucose and 2-hour postprandial blood glucose were lower in the almond group compared to the control group, although the differences were not significant (*p* > 0.05). The mean differences were −0.03 (95% CI: −0.18, 0.11) for fasting blood glucose and −0.15 (95% CI: −0.44, 0.13) for postprandial blood glucose.

The sensitivity analysis conducted by removing studies one by one from the meta-analysis did not change the results in relation to HbA1c (*p* < 0.05), fasting blood glucose (*p* > 0.05), and 2 h postprandial blood glucose (*p* > 0.05). The sub-group analysis for HbA1c showed that, although there was a significant difference (*p* < 0.001) between the almond group and control with respect to the meta-analysis of the randomised parallel studies, the differences were not significant (*p* = 0.25) in relation to the cross-over studies (Figure 3b).

### 4.4. Inflammatory Markers

The study by Liu et al. [34] observed that the addition of almonds into a healthy diet could ameliorate inflammation and oxidative stress in patients with type 2 diabetes. Similarly, Sweazea et al. [36] noted that the daily consumption of almonds in the absence of other dietary or physical activity activities could be an effective approach in reducing inflammation in patients with type 2 diabetes.

**Figure 4:**
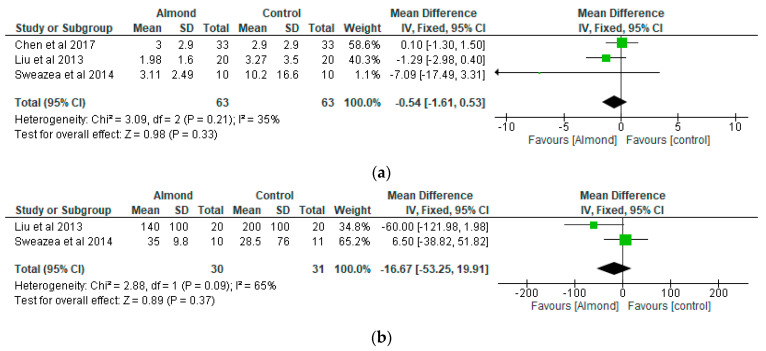
The effect of almonds on (**a**) C-reactive protein (mg/L) and (**b**) tumour necrosis factor–α (pg/mL).

The meta-analyses of the effects of almonds on inflammatory markers are shown in Figure 4a,b. Three studies contributed data for the C-reactive protein analysis (almond gp, *n* = 63; control gp, *n* = 63) (Figure 4a) and two studies for tumour necrosis factor- α (TNF- α) analysis (almond gp, *n* = 30; control gp, *n* = 31) (Figure 4b). The levels of C-reactive protein and TNF- α were lower in the almond group compared to the control group. However, the differences between the two groups were not significant (*p* > 0.05), with mean differences of −0.54 (95% CI: −1.61, 0.53) for C-reactive protein and −16.67 (95% CI: −53.25, 19.91) for TNF- α respectively. The results did not change between the almond group and control group (*p* > 0.05) with respect to C-reactive protein and TNF- α following sensitivity tests.

### 4.5. Body Mass Index (BMI) (Kg/m^2^)

Chen et al. [31] did not find any significant effect of the almond-based diet with respect to body weight and BMI. In contrast, Cohen et al. [32] found that chronic almond ingestion resulted in a 4% reduction in BMI compared with control (*p* = 0.047). Six studies contributed to the results of the analysis for BMI (almond gp, *n* = 105; control gp, *n* = 105).

**Figure 5:**
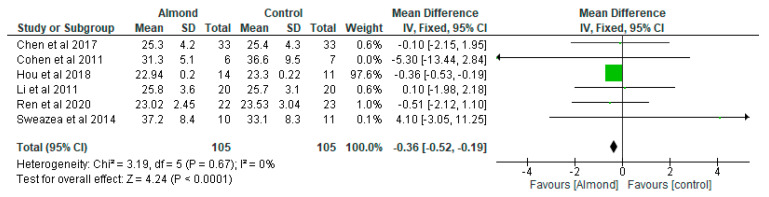
The effect of almonds on body mass index (Kg/m^2^).

The meta-analysis showed that the BMI was significantly lower (*p* < 0.05) in the almond group compared with the control group (Figure 5), with a mean difference of −0.36 (95% CI: −0.52, −0.19). The results of the sensitivity analysis showed consistency in terms of the significant difference between the almond group and the control group, except when the Hou et al. [6] study was removed.

### 4.6. Homeostatic Model Assessment of Insulin Resistance (HOMA–IR)

According to Chen et al. [31], the almond-based diet did not show a significant effect with respect to HOMA-IR compared with control.

**Figure 6:**
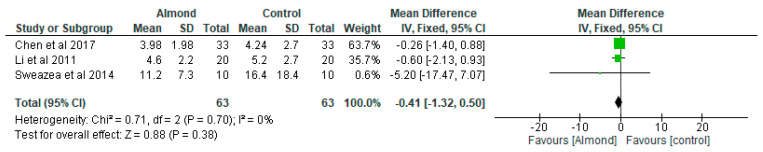
The effect of almond on HOMAR–IR.

Three studies contributed data for HOMA-IR meta-analysis (almond gp, *n* = 63; control gp, *n* = 63) and the difference between the almond and control groups was not significant (*p* > 0.05) (Figure 6). The mean difference was −0.41 (95% CI: −1.32, 0.50). The sensitivity analysis did not change the results between the almond group and the control group (*p* > 0.05) in respect of HOMA–IR.

### 4.7. Glucagon-Like Peptide-1 (GLP-1)

There was significant difference (*p* < 0.05) between the almond-based diet group and the control group with respect to the GLP-1 in the study by Ren et al. [14], although Cohen et al. [32] did not find any significant differences (*p* > 0.05) between the two groups.

**Figure 7:**
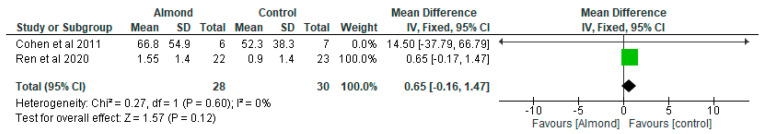
The effect of almond on GLP-1 (pmol/L).

Regarding the GLP-1 meta-analysis, two studies contributed data (almond gp, *n* = 28; control gp, *n* = 30) (Figure 7). GLP-1 was higher in the almond-based diet group compared with control, although the difference was not statistically significant (mean difference: 0.65; 95% CI: −0.16, 1.47; *p*-value = 0.12). The sensitivity analysis did not change the results between the almond group and the control group (*p* > 0.05) with to respect to GLP-1.

### 4.8. Fasting Insulin

**Figure 8:**
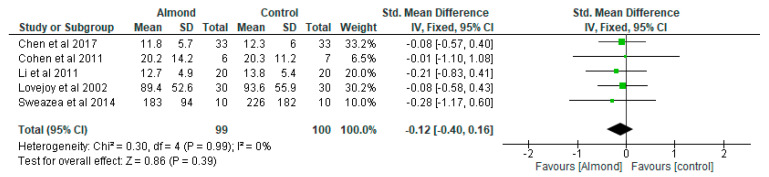
The effect of almond on fasting insulin (standardised mean difference).

Bodnaruc et al. [30] found that an almond-based diet was associated with lower insulinemia, while Chen et al. [31] did not find any significant effect with respect to insulin levels in the almond-based and control diets. Five studies contributed data for this outcome (almond gp, *n* = 99; control gp, *n* = 100) (Figure 8). It was observed that there was no significant difference between the almond-based group compared to the control group in relation to insulin (standardised mean difference: −0.12; 95% CI: −0.40, 0.16; *p*-value = 0.39). There was also no significant difference (*p* > 0.05) between the almond group and the control group following the sensitivity analysis in regards to fasting insulin.

## 5. Discussion

The results of the systematic review suggest that almond-based diets can promote the growth of short-chain fatty acid (SCFAs)-producing gut microbiota. Furthermore, the meta-analysis showed that almond-based diets were effective in significantly lowering (*p* < 0.05) glycated haemoglobin and body mass index (BMI) in patients with type 2 diabetes. However, it was also found that the effects of almond-based diets were not significant (*p* > 0.05) in relation to fasting blood glucose, 2 h postprandial blood glucose, inflammatory markers (C-reactive protein and TNF-α), GLP-1, HOMA–IR, and fasting insulin.

Our findings of the beneficial effects of almond-based diets on glycated haemoglobin are consistent with a previous study on almond supplementation in patients with type 2 diabetes [19] and an earlier review on the effect of tree nuts on glycaemic control in patients with diabetes [22]. Similarly, our results in relation to BMI are consistent with the findings of a previous study on the effect of almond consumption in the general population [18] and an earlier review of the effect of almonds on BMI [37].

The biological mechanisms responsible for the outcomes observed in this review in relation to reduction in glycated haemoglobin and BMI may be based on the nutrient composition of almond and its biological effects [37]. When compared to other nuts, it has been reported that almonds have the highest levels of fibre, monounsaturated and polyunsaturated fats, flavonoids, phytosterols, and phenolic acids [5,37]. Almonds also have a low glycaemic index [5] and almond-based diets have been shown to modulate gut microbiota dysbiosis and promote the production of GLP-1 in patients with type 2 diabetes [14].

The glycaemic index (GI) of food is an important measure of the quality of the food and it is a reflection of the digestibility of the available carbohydrates in the food compared with the reference food, often glucose [38]. It is a measure that ranks food based on the blood glucose response that they produce when ingested compared with the response to glucose or white wheat bread, which are reference foods [39]. Therefore, foods with low GI, such as almonds, usually breakdown slowly during digestion, and are slowly assimilated and, thus, have a slower impact on blood glucose levels and insulin response [40,41,42]. In a previous systematic review and meta-analysis, Ojo et al. [40], found that diets with low GI were more effective in improving glycated haemoglobin and fasting blood glucose compared with high-GI diets in patients with type 2 diabetes. In contrast, diets with high GI have been associated with type 2 diabetes and cardiovascular diseases due to their effect on blood glucose and insulin levels [38].

Due to the gradual entrance of glucose into the blood leading to reduced and more sustained insulin release, low-GI diets are more effective in controlling glycaemia compared with high GI diets [41]. In addition, low GI diets may be effective in increasing insulin sensitivity by reducing fluctuations in blood glucose levels and minimising insulin secretion over the day [41]. Based on the effectiveness of low-GI diets in controlling glycemia in patients with diabetes, the FAO [42] has recommended the use of a glycaemic index of foods along with the information about food composition in clinical applications in patients with diabetes.

Apart from the potential to improve glycaemic control, it has been suggested that diets with low GI may be useful in reducing weight because they produce a low insulin response [43]. This view is based on the lipogenic effect of hyperinsulinaemia [43]. On the other hand, high-GI diets may elicit a higher postprandial insulin response and this may lead to quicker hunger response and overeating through the reduction in metabolic fuels in the body [43]. Increased satiety and reduced voluntary food intake has been proposed as another mechanism through which foods with low GI can reduce weight [43].

Nuts, including almonds, are rich in energy density and high in fat, therefore, the greater fat availability could lead to reduced gastric emptying and increased satiety [5,14].

Another area of interest is the high soluble fibre and unsaturated fatty acid content of almonds [6]. According to Huo et al. [6], unsaturated fatty acids have been reported to promote the movement of glucose receptors to the cell surface and this could enhance insulin sensitivity. The role of polyunsaturated fatty acids on insulin sensitivity may be based on the fatty acid composition of the cell membrane, which relies on the fatty acid composition of the diet and regulates insulin action [44]. Kien et al. [45] suggested that a possible mechanism of dietary fatty acids in reducing the risk of insulin resistance may be due to the presence of a high level of unsaturated fatty acids in the cell membrane that could influence the physical properties, including plasticity, which promotes the movement of glucose receptors to the cell surface. It has also been shown that skeletal muscle insulin resistance due to obesity or dietary fatty acids may result from defective mitochondrial oxidation of fatty acids, which could lead to the accumulation of ceramides that may inhibit insulin signalling [45]. In addition, a high saturated fatty acid level of the membrane phospholipids increases insulin resistance [44].

Haag and Dippenaar [46] noted that the high saturated fat content of the cell membrane may lead to rigid and unresponsive membranes, while membranes that are high in unsaturated fatty acids promote fluidity and responsiveness. Therefore, the polyunsaturated content and omega-3/omega-6 ratio in the muscle and fat membranes are of significant importance in the aetiology of insulin resistance [46]. Furthermore, fatty acid-derived entities such as long chain acyl-CoA (coenzymes) may impact negatively on insulin mediated glucose transport and disrupt the insulin signalling cascade [46]. These findings were confirmed in randomised controlled trials in overweight individuals conducted by Kahleova et al. [47], who found that fat quantity and quality were related to body weight and body composition, insulin secretion, and insulin resistance.

Unsaturated fatty acids can also promote the efficiency of β-cell function through their action in stimulating GLP-1 secretion [6]. The findings of this review did reveal that an almond-based diet was effective in promoting the secretion of GLP-1, although this was not significant compared to the control. GLP-1 is a 30-amino-acid agent, which regulates glucose by stimulating insulin after ingesting a meal [32].

High dietary fibre in almonds can also increase gastric distension, viscosity of gastrointestinal tract, and slower absorption of macronutrients, including slowing the absorption of carbohydrates and the level of postprandial blood glucose [6]. High dietary fibre has been reported to promote the growth of SCFAs producing bacteria, increasing the production of SCFAs and promoting GLP-1 secretion [14].

In the study by Zhao et al. [48], it was found that the presence of greater diversity and abundance of fibre-promoting SCFA producers improved glycated haemoglobin levels in patients with type 2 diabetes through the production of glucagon-like peptide-1. The dietary fibre undergoes fermentation by colonic microbiota to produce SCFAs, including propionic, acetic, and butyric acid, which have significant effects on host physiology [49]. The SCFAs are useful in regulating the metabolic and immune system of the host as well as in cell proliferation [50]. However, low dietary fibre intake can cause microbiota dysbiosis, reduction in SCFAs production, and lead to the utilisation of less favourable substrates, such as proteins and fat [50,51]. The lipopolysaccharides resulting from the use of a high-fat diet can elicit an inflammatory response and contribute to the development of insulin resistance and type 2 diabetes [51].

### Limitations

One of the studies included [32] was a pilot study with a small sample size. Furthermore, the number of studies included in the meta-analyses was eight or smaller in the different parameters. These could affect the broader application of the findings of the review. Therefore, more studies are required to further explore the role of almonds in patients with type 2 diabetes.

## 6. Conclusions

The findings of this systematic review and meta-analysis have shown that almond-based diets may be effective in promoting short-chain fatty acid-producing bacteria, and lowering glycated haemoglobin and body mass index in patients with type 2 diabetes compared with control. However, the effects of almonds were not significant (*p* > 0.05) with respect to fasting blood glucose, 2 h postprandial blood glucose, inflammatory markers (C-reactive protein and TNF-α), GLP-1, HOMA–IR, and fasting insulin.

## Figures and Tables

**Table 1:** Search terms and search strategy.

Patient/Population	Intervention	Comparator	Outcome (Primary)	Study Designs	Combining Search Terms
Patients with diabetes	Almonds	Control	Glycometabolism	Randomised controlled trial	
Patients with diabetes OR type 2 diabetes OR Diabetes OR Diabetes complications OR diabetes mellitus, type 2 OR diabetes mellitus	Almond OR Tree, Almond OR Almond Tree OR Sweet Almond OR Almond Trees OR Tree Nuts OR Almond, Sweet			1. Randomised controlled trial OR controlled clinical trial OR randomized OR placebo OR drug therapy OR randomly OR trial OR groups 2. “Animals” NOT “Humans” 3. 1 NOT 2	Column 1 AND Column 2 AND Column 3

**Table 2:** General characteristics of included studies.

Citation/Country of Study	Type of Study	Sample Details and Duration of Study	Mean Age (Years)	Aim	Interventions	Results
Bodnaruc et al. [30] Canada	A randomised cross-over study	7 men with type 2 diabetes. Data were collected during two experimental sessions separated by a ≥7day washout period.	63.9 ± 2.5	To evaluate the effects of almonds on postprandial glucose response.	Participants completed 2 experimental visits and control (white bread, butter, cheese) and test (white bread, almonds) diets were ingested.	The test meal was associated with lower postprandial glycemia and insulinemia.
Chen et al. [31] Taiwan	A randomised cross-over controlled study	40 patients with type 2 diabetes. 12-weeks duration.	54.9 ± 10.5	To examine the effect of almonds on glycaemia	Approximately 60 g/day almonds added to a National Cholesterol Education Programme Step II diet (NCEP II) compared to NCEP II diet alone as control	Both almond-based and control diets did not significantly affect body weight and BMI or change HbA1c, fasting serum glucose, insulin, or HOMA-IR values.
Cohen et al. [32] USA	A randomised parallel study	13 participants diagnosed with type 2 diabetes Almond-based diets (*n* = 6) Control (*n* = 7) 12-weeks duration.	Almond group: 66 ± 3.3 Control group: 66 ± 3.3	To examine the impact of chronic almond ingestion on glycaemic control in patients with type 2 diabetes.	Participants were randomised to almond group (1 oz of almonds, 5 days/week) or cheese group (2 cheese sticks, 5 days/week)	HbA1c was the only blood marker that changed significantly between the treatment groups (*p* = 0.045). Chronic almond ingestion resulted in a 4% reduction in BMI compared with control (*p* = 0.047).
Hou et al. [6] China	A randomised controlled study	Almond group (*n* = 14) Peanut group (*n* = 11) 12-weeks duration.	Almond group: 70.86 ± 8.21 Peanut group: 68 ± 5.80	To compare the effects of peanuts and almonds incorporated into a low-carbohydrate diet on cardiometabolic and inflammatory parameters in patients with type 2 diabetes	Peanuts or almonds were incorporated into a low-carbohydrate diet and both groups were compared after a 3-month intervention.	Almonds and peanuts have similar effect on improving fasting and postprandial blood glucose among patients with type 2 diabetes when incorporated into a low-carbohydrate diet.
Li et al. [33] Taiwan	Randomised cross-over clinical trial	20 Chinese patients with type 2 diabetes. 12-weeks duration.	58 ± 2	To evaluate the effect of almond consumption on glycaemia in Chinese patients with type 2 diabetes	Incorporation of almonds into National Cholesterol Education Programme Step II diet (NCEP II) to replace 20% of total daily calorie intake compared with NCEP II diet alone as control.	Adding almonds into a healthy diet has beneficial effects on adiposity and glycaemic control.
Liu et al. [34] Taiwan	Randomised cross-over controlled feeding trial	20 Chinese patients with type 2 diabetes. 12-weeks duration.	58 ± 2	To examine the effect of almond consumption on inflammation and oxidative stress in patients with type 2 diabetes	Addition of almonds (approximately 56 g/day) into National Cholesterol Education Programme Step II diet (NCEP II) to replace 20% of total daily calorie intake compared with NCEP II diet alone as control.	Adding almonds into a healthy diet could ameliorate inflammation and oxidative stress in patients with type 2 diabetes.
Lovejoy et al. [35] USA	Randomized double-blind crossover design	30 participants with type 2 diabetes. 16-weeks duration.	53.8 ± 1.9	To assess the effects of almond-enriched diets on insulin sensitivity in patients with diabetes	The 4 diets were as follows: (1) high-fat, high-almond (HFA; 37% total fat, 10% from almonds); (2) low-fat, high-almond (LFA; 25% total fat, 10% from almonds); (3) high-fat control (HFC; 37% total fat, 10% from the MUFAs from olive or canola oil); and (4) low-fat control (LFC; 25% total fat, 10% from olive or canola oil). The almond-containing diets provided 57–113 g almonds/d depending on the total energy level	Almond-enriched diets did not influence glycaemia in patients with diabetes.
Ren et al. [14] China	Randomised controlled trial	45 participants with type 2 diabetes. 12-weeks duration.	LCD group: 73.55 ± 4.99 LFD group: 70.48 ± 5.91	To determine the effect of almond-based low-carbohydrate diet on glycometabolism, gut microbiota, and GLP-1 in patients with type 2 diabetes.	The intervention group consumed a low-carbohydrate diet, which included 56 g/day almonds that replaced 150 g/day staple food, while the control group adopted a low-fat diet education programme.	Almond-based LCD may be effective in regulating glycometabolism in patients with diabetes by stimulating the growth of SCFA-producing bacteria, increasing SCFA production and promoting GLP-1 secretion. The almond-based LCD significantly increased the SCFA-producing bacteria *Roseburia*, *Ruminococcus,* and *Eubacterium*.
Sweazea et al. [36] USA	Randomised controlled study	21 participants with type 2 diabetes. 12-weeks duration.	Almond group: 57.8 ± 5.6 Control group: 54.7 ± 8.9	To evaluate if almond supplementation without further dietary advice improves glycaemic control compared with control.	The almond group consumed 43 g almonds 5–7 times per week and to maintain their usual diet and activity pattern while the control group maintained their usual diet and activity pattern.	Daily almond consumption in the absence of other dietary or physical activity activities is useful in reducing inflammation in patients with type 2 diabetes.

Abbreviations: LCD, low-carbohydrate diet; LFD, low-fat diet; GLP-1, glucagon-like peptide-1; MUFAs, monounsaturated fats; SCFAs, short-chain fatty acid.

## Data Availability

Not applicable.
